# Genotype-Based Ancestral Background Consistently Predicts Efficacy and Side Effects across Treatments in CATIE and STAR*D

**DOI:** 10.1371/journal.pone.0055239

**Published:** 2013-02-06

**Authors:** Daniel E. Adkins, Renan P. Souza, Karolina Åberg, Shaunna L. Clark, Joseph L. McClay, Patrick F. Sullivan, Edwin J. C. G. van den Oord

**Affiliations:** 1 Center for Biomarker Research and Personalized Medicine, School of Pharmacy, Medical College of Virginia, Virginia Commonwealth University, Richmond, Virginia, United States of America; 2 Departments of Genetics, Psychiatry, & Epidemiology, University of North Carolina at Chapel Hill, North Carolina, United States of America; 3 Department of Medical Epidemiology & Biostatistics, Karolinska Institutet, Stockholm, Sweden; National Taiwan University, Taiwan

## Abstract

Only a subset of patients will typically respond to any given prescribed drug. The time it takes clinicians to declare a treatment ineffective leaves the patient in an impaired state and at unnecessary risk for adverse drug effects. Thus, diagnostic tests robustly predicting the most effective and safe medication for each patient prior to starting pharmacotherapy would have tremendous clinical value. In this article, we evaluated the use of genetic markers to estimate ancestry as a predictive component of such diagnostic tests. We first estimated each patient’s unique mosaic of ancestral backgrounds using genome-wide SNP data collected in the Clinical Antipsychotic Trials of Intervention Effectiveness (CATIE) (n = 765) and the Sequenced Treatment Alternatives to Relieve Depression (STAR*D) (n = 1892). Next, we performed multiple regression analyses to estimate the predictive power of these ancestral dimensions. For 136/89 treatment-outcome combinations tested in CATIE/STAR*D, results indicated 1.67/1.84 times higher median test statistics than expected under the null hypothesis assuming no predictive power (*p*<0.01, both samples). Thus, ancestry showed robust and pervasive correlations with drug efficacy and side effects in both CATIE and STAR*D. Comparison of the marginal predictive power of MDS ancestral dimensions and self-reported race indicated significant improvements to model fit with the inclusion of MDS dimensions, but mixed evidence for self-reported race. Knowledge of each patient’s unique mosaic of ancestral backgrounds provides a potent immediate starting point for developing algorithms identifying the most effective and safe medication for a wide variety of drug-treatment response combinations. As relatively few new psychiatric drugs are currently under development, such personalized medicine offers a promising approach toward optimizing pharmacotherapy for psychiatric conditions.

## Introduction

It is well-known that only a subset of patients will respond to any given prescribed drug [1]. The time it takes a clinician to declare a treatment ineffective leaves the patient in an impaired state and at unnecessary risk for adverse drug effects. Furthermore, drug nonresponse reduces the likelihood of compliance and adherence to future treatments [2]. Therefore, diagnostic tests capable of identifying the most effective and safe medication for each patient prior to initiating pharmacotherapy would have tremendous clinical value [3], [4]. Predicting drug nonresponse has, however, proven to be difficult. These challenges have led to a proliferation of pharmacogenetics research in the last decade. This research has traditionally focused on pharmacodynamic and pharmacokinetic candidate genes that encode drug targets or are involved in the metabolism of the drug itself. More recently, genome-wide association studies (GWAS) systematically screening markers across the whole genome for association with drug response have been added as a tool to identify relevant genetic variants [5]. However, before these genetic markers can be used in the clinic, they will need to be evaluated more extensively through replicated association and functional studies.

In the absence of firmly established panels of genetic markers predicting the effects of specific drugs, it is sensible to search for proxy variables robustly capturing relevant genetic differences between individual patients. These proxies could serve as interim components in the development of predictive algorithms for individualizing pharmacotherapy. Based on observations of variability in drug-response between populations [6], [7], [8], we hypothesize that ancestry information could be one such proxy. To evaluate this hypothesis we used clinical and genetic information from the two largest psychiatric clinical trials to test therapy efficacy conducted in the United States: the Clinical Antipsychotic Trials of Intervention Effectiveness (CATIE) [9] (ClinicalTrials.gov Identifier: NCT00014001) and the Sequenced Treatment Alternatives to Relieve Depression (STAR*D) [10] (ClinicalTrials.gov Identifier: NCT00369746). Ancestral dimensions were derived from genome-wide arrays including differences across hundreds of thousands of single-nucleotide polymorphisms (SNPs).

## Methods

For both the CATIE and STAR*D studies, Supporting Information S1 provides detailed information about the subjects and study design, assessment instruments, estimation of treatment effects, genotyping and estimation of ancestral dimensions. We restrict ourselves to a short description here.

The CATIE study participants were recruited from 57 clinical settings around the United States [9], [11]. The Structured Clinical Interview for DSM-IV was used to establish schizophrenia diagnosis. The study consisted of a baseline, three phases and a follow up. Patients were typically switched to another drug because of a lack of efficacy or adverse effects. The STAR*D study is a prospective, randomized clinical trial of outpatients with non-psychotic major depressive disorder [10], [12]. The Structured Clinical Interview for DSM-IV was used to establish non-psychotic major depressive disorder diagnosis. Sample collection involved 41 clinical sites across the United States. The full clinical trial study sample includes 4,000 adults from both primary and specialty care practices who had shown neither inadequate response nor intolerance to any of the protocol treatments. The study consisted of four phases. In the first phase all patients started with citalopram. Different medications or medication combinations for treatment resistant subjects were administered in each subsequent phase.

Table 1 shows, for the main drug and outcome measures in CATIE and STAR*D, the number of subjects assessed and the mean number of observations across the entire trial. For clozapine in CATIE, the sample sizes (∼50) were much more modest than the other drugs, for which there were on average 218 subjects per drug-outcome combination with 3.6 assessments for each subject. Citalopram in STAR*D had much higher sample sizes (1870) and number of assessments (4.6) than the other antidepressants, which had an average of 127 subjects per drug-outcome combination with 3.8 assessments per subject.

**Table 1:** Number of subjects assessed and mean number of observations for each treatment-phenotype comparison. We present only a summary of phenotypes tested in this study.

Study/Treatment	Phenotype															
CATIE	PANSS total		PANSS positive		PANSS negative		Neuro-cognitive		Body mass index		Total cholesterol		QTc prolongation		Heart rate	
	N	Obs	n	Obs	n	Obs	n	Obs	n	Obs	n	Obs	n	Obs	n	Obs
Perphenazine	130	4.68	130	4.68	130	4.68	130	4.68	145	4.19	142	2.52	121	1.65	144	4.18
Clozapine	46	3.83	46	3.83	46	3.83	46	3.83	52	3.37	53	2.21	51	1.92	52	3.35
Olanzapine	254	4.86	254	4.86	254	4.86	254	4.86	283	4.33	281	2.69	249	1.59	282	4.35
Quetiapine	238	3.98	238	3.98	238	3.98	238	3.98	277	3.40	266	2.26	224	1.51	278	3.40
Risperidone	254	4.33	254	4.33	254	4.33	254	4.33	282	3.91	272	2.47	239	1.55	280	3.92
Ziprasidone	168	3.63	168	3.63	168	3.63	168	3.63	200	3.00	195	1.97	189	1.62	198	3.01
STAR*D	**QIDS Self-report**		**QIDS Clinician**		**Depressed affect**		**Insomnia**		**Appetite/Weight**		**Dizziness**		**Sex**		**Eyes/Ears**	
	**n**	**Obs**	**n**	**Obs**	**n**	**Obs**	**n**	**Obs**	**n**	**Obs**	**n**	**Obs**	**n**	**Obs**	**n**	**Obs**
Citalopram	1867	4.93	1892	5.07	1860	4.92	1879	4.92	1848	4.93	1864	4.07	1891	4.06	1862	4.06
Bupropion	138	3.61	139	3.7	138	3.61	139	3.61	137	3.61	135	3.69	138	3.69	135	3.69
Citalopram+Bupropion	169	4.04	172	4.16	169	4.03	169	4.02	168	4.03	172	4.14	172	4.12	172	4.14
Citalopram+Buspirone	160	3.86	164	4.01	159	3.86	160	3.85	159	3.85	163	4.02	164	4.01	163	4.02
Mirtazapine	69	3.36	70	3.44	69	3.34	69	3.34	67	3.34	68	3.46	70	3.44	68	3.46
Nortriptyline	73	3.32	75	3.41	73	3.29	73	3.29	72	3.29	75	3.41	75	3.41	75	3.41
Sertraline	123	3.86	128	4.02	121	3.85	124	3.84	121	3.84	125	3.97	128	3.96	125	3.96
Venlafaxine	156	3.92	156	4.1	154	3.91	156	3.9	152	3.91	152	4.04	156	4.05	151	4.04

PANSS – Positive and Negative Syndrome Scale. QTc - QT interval corrected for heart rate. QIDS – Quick Inventory of Depressive Symptomatology.

In CATIE, 665,439 SNPs were genotyped using the Affymetrix 500 K chipset (Santa Clara, CA, USA) and a custom 164 K chip created by Perlegen (Mountain View, CA, USA). After quality control, genotypes for 492,900 SNPs from 738 individuals remained for investigating ancestral background dimensions [13]. In STAR*D a total of 969 subjects were genotyped at Affymetrix, Inc. (South San Francisco) on the Human Mapping 500 K Array Set and another 979 samples were genotyped using the Affymetrix Genome-Wide Human SNP Array 5.0. The two groups were balanced by ethnic grouping, gender and proportions of responders and non-responders. Twelve samples were genotyped on both the 500 K and 5.0 Arrays, with a >99% concordance across these platforms [14]. After QC, 430,198 SNPs remained for use in the current analysis of ancestral background dimensions.

To estimate ancestral background dimensions, we used the multi-dimensional scaling (MDS) approach implemented in PLINK [15], which has been demonstrated to be essentially equivalent to the principal component method implemented in EigenSoft [16]. Input data for the MDS approach were the genome-wide average proportion of alleles shared identical by state between any two individuals. The first ancestral dimension captures the maximal variance in the genetic similarity; the second dimension must be orthogonal to the first and captures the maximum amount of residual genetic similarity; and so on. The first five dimensions appeared to capture the vast majority of ancestral variation in the CATIE and STAR*D samples and they were used in the current analysis. The same number of dimensions used here have been used in previous analyses of CATIE and STAR*D [17], [18], [19], [20], [21], [22], [23], [24].

One important, often neglected, issue in genomic studies using genotype array-based estimates of ancestral background dimensions (i.e., population structure) is the fact that various technical genotyping artifacts can give rise to artifactual variance in array data, which may in turn be captured as spurious ancestral dimensions. These technical genotyping artifacts have the potential to cause false positive associations if they are correlated with the phenotypic outcome. Thus, for instance, in a case-control study where cases and controls were genotyped in separate runs, without rigorous QC for potential batch and plate effects, there would be a serious risk that observed association between case-control status and “ancestral dimensions” would actually be driven by genotyping artifacts.

However, in the current study there are multiple sources of evidence precluding the possibility of false positives due to genotyping artifacts. The strongest evidence comes from the fact that correlations between any genotyping artifacts and treatment response are virtually impossible here. This is because in STAR*D each array was, “balanced by ethnic grouping, sex, and proportions of responders and nonresponders” [25]. For CATIE, among the schizophrenia cases examined here, batch and plating was randomized with no knowledge of treatment response status [13]. Thus, correlations between batch (or plate) effects and treatment response are explicitly impossible due to careful design in STAR*D, and highly unlikely CATIE, as they would entail a significant association between treatment response and a random variable with no correlated signal (randomized plating and batch assignment). These array randomization/balancing procedures also essentially eliminate the possibility of correlation between treatment response and other array-based artifacts, including systematic subject differences in call rate and proportion of allelic heterozygosity.

As an added precaution, however, both studies were QC’ed for heterozygosity and call rate per subject (along with numerous other rigorous QC procedures described in the original studies and their supplemental materials [13], [25]). Thus, in the post-QC data analyzed here, call rates for all subjects were stringently high (<.99.2) and all heterozygosity rates fell within ±3 SD of the mean, the threshold proposed in standard GWAS QC guidelines [26]. Thus, due to balanced/randomized batch and plating and rigorous QC, the risk of systematic genotype errors causing false positives is effectively eliminated. However, it remains possible that nonsystematic (i.e., uncorrelated with treatment response) genotyping errors may have eluded QC procedures and, thus, contribute to the variance of the ancestral dimensions. However, in this worst case scenario, artifactual variance would produce noise in the ancestral dimensions, increasing the risk of false negative associations. Thus, results of the current analysis may be considered conservative.

We previously developed a systematic method to estimate treatment effects [27]. Our method uses mixed models to first estimate the optimal functional form of over-time drug response, then screens many possible covariates to select those that improve the precision of the treatment effect estimates, and finally generates individual treatment effect estimates based on the best fitting model using best linear unbiased predictors (BLUPs) [28]. As our approach condenses all information collected during the trials in an optimal, empirical fashion, it results in more precise estimates than traditional approaches (e.g., subtracting pre- from post-treatment observations) that estimate treatment effects using only two assessments. We have successfully applied this method in several genome-wide association studies performed on CATIE and STAR*D samples [17], [18], [19], [20], [21], [22], [23], [29].

After estimating MDS dimensions and treatment effects, we performed multiple regressions to evaluate the association of MDS dimensions and/or self-reported ethnicity with each drug-outcome treatment response combination. These analyses were conducted to investigate two specific issues. First, we considered whether genotype-based ancestry has consistent, significant prognostic power in predicting psychiatric drug response, and if so, how strong is this predictive power. Second, we examined whether genotype-based ancestry significantly improved prediction of psychiatric drug response *over and above* the predictive power of self-reported ethnicity.

In investigating the first issue, we analyzed the distribution of F-tests of model fit for the 5 MDS prediction models, as well as summarizing the individual drug coefficients, multiple correlations and number of significant models adjusting for multiple testing [30], [31]. To address the second issue, we compared differences in multiple correlations and number of significant models between full models (containing both self-reported ethnicity and MDS dimensions) and nested reduced models excluding either self-reported-ethnicity or MDS dimensions. These comparisons describe the marginal contribution of the MDS variables and self-reported ethnicity, respectively. To formally test the statistical significance of these marginal effects, we conducted F-tests of model fit between the full and reduced models. In addition to summarizing the number and proportion of models in which the MDS dimension had significant explanatory power over and above self-reported ethnicity, we also performed Chi-squared tests of proportions to determine if the number of significant marginal effects for the MDS variables was more than expected by chance. For comparison, the statistical significance of self-reported ethnicity marginal effects was likewise analyzed.

Finally, after establishing the value of GWAS-based ancestral dimensions as predictors of psychiatric drug response, we then empirically demonstrate that much smaller sets of markers can be used to capture this ancestral information. This exercise serves as proof of concept that such an approach could be applied in clinical settings using small, inexpensive genotype arrays. Further, we provide our empirically determined SNP lists and the weights used to calculate these proxy MDS dimensions in Supporting Information S2, as a resource for researchers interested in replicating these findings or extending efforts to develop predictive algorithms of psychiatric drug response.

## Results

### Predictive Power of Genotype-based Ancestry

**Figure 1:**
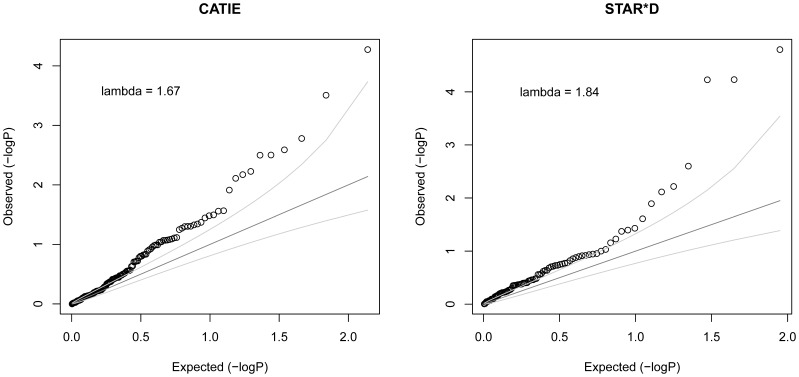
Quantile-quantile (Q-Q) plots for the joint effects of MDS dimensions on various drug response measures. Points represent ordered model fit F-test -log_10_(*p-*values), each of which quantifies the explanatory power of a model of 5 MDS ancestral dimensions predicting a measure of treatment response. The straight, dark grey lines represent the expected *p*-value distribution under the null hypothesis of no true associations. Light grey lines represent 95% confidence intervals for rejecting the null hypothesis at each *p*-value rank. The inflation parameter, lambda, is defined as the ratio of the median observed test statistic to the expected median under the null distribution; thus, lambda quantifies the degree to which the test statistic distribution systematically diverges from the null expectation of no significant effects.

Figure 1 summarizes results of the regression analyses to test the null hypothesis that the five ancestral dimensions do not predict drug response (i.e., observed association is due to statistical noise and not true signal) using a Quantile-Quantile (QQ) plot for each of the drug-outcome combinations. The ordered, observed model fit F-test *p*-values are plotted against those expected under the null hypothesis of no true associations among the 136 (CATIE) or 89 (STAR*D) tests, represented by the straight line. The QQ plots show that the observed *p*-values deviated systematically from this straight line and were well outside the 95% confidence intervals. This provides strong evidence that the five ancestral dimensions systematically and significantly predicted efficacy and adverse reaction across these psychiatric pharmacotherapies.

To more exactly quantify the degree to which the ancestral dimensions predicted drug response, we calculated the ratio of the median observed test statistic to the expected test statistic under the null hypothesis. This ratio is commonly used in GWAS as a measure of the degree to which associations are due to population differences, and is denoted as lambda (λ) [16], [32]. In the current context, λ >1 suggests that the ancestral differences captured by the MDS dimensions do, in fact, influence psychiatric treatment response. Lambda values were calculated as 1.67 and 1.84 for CATIE and STAR*D, respectively. Thus, the median of model fit test statistics was 1.67 and 1.84 times higher than expected under the null hypothesis for CATIE and STAR*D, respectively. One-sample Wilcoxon signed rank tests of the median (CATIE: V-statistic = 3323, p-value <0.01; STAR*D: V-statistic = 1097, *p*-value <0.001) confirmed that these test statistics were systematically larger, and *p*-values smaller, than expected by chance.

A summary of the predictive power of the ancestral dimensions on individual drug-outcome combinations shows that ancestry explained a nontrivial portion of variance in both drug efficacy and side effect outcomes across all treatment regimens (Table 2). The mean (multiple regression) correlation coefficient was 0.19 in CATIE and 0.20 in STAR*D, suggesting the 5 dimensions explained on average about 3.7% and 4.0% of the variation in antipsychotic and antidepressant response, respectively.

**Table 2:** Summary of the average multiple regression correlation coefficients for the treatment-outcome combinations.

Study/Treatment	Phenotype							
CATIE	PANSS total	PANSS positive	PANSSnegative	Neuro-cognitive	Body mass index	Total cholesterol	QTc prolongation	Heart rate
Perphenazine	0.174	0.172	0.193	0.283	0.093	0.073	0.094	0.145
Clozapine	0.206	0.174	0.236	0.229	0.429	0.352	0.422	0.346
Olanzapine	0.102	0.131	0.116	0.127	0.202	0.147	0.142	0.134
Quetiapine	0.161	0.168	0.107	0.238	0.293	0.151	0.159	0.207
Risperidone	0.136	0.081	0.101	0.242	0.114	–	0.078	0.031
Ziprasidone	0.180	0.197	0.142	0.213	0.131	0.141	0.100	0.068
STAR*D	**QIDS Self-report**	**QIDS Clinician**	**Depressed affect**	**Insomnia**	**Appetite/Weight**	**Dizziness**	**Sex**	**Eyes/Ears**
Citalopram	0.126	0.119	0.120	0.062	0.052	0.099	0.031	0.036
Bupropion	0.205	0.158	0.192	0.221	0.180	0.163	0.121	0.164
Citalopram+Bupropion	0.290	0.304	0.302	0.210	0.138	0.194	0.175	0.210
Citalopram+Buspirone	0.156	0.231	0.200	0.191	0.186	0.173	0.072	0.206
Mirtazapine	0.306	0.233	0.334	0.353	0.364	0.334	0.206	0.274
Nortriptyline	0.280	0.337	0.280	0.229	0.417	0.321	0.141	0.305
Sertraline	0.274	0.240	0.272	0.074	0.131	0.173	0.144	0.200
Venlafaxine	0.176	0.125	0.157	0.228	0.227	0.141	0.145	0.185

PANSS – Positive and Negative Syndrome Scale. QTc - QT interval corrected for heart rate. QIDS – Quick Inventory of Depressive Symptomatology.

### Genotype-based Ancestry and Self-reported Ethnicity

To further study the derived MDS dimensions, we present the correlations of the five MDS dimensions with self-reported ethnicity (European American, African American and Hispanic) in Supporting Information S1. Results showed that MDS 1 generally captured ancestral differences related to European and African American ancestry, while MDS 2 and 3 seemed to capture differences between Hispanic v. non-Hispanics groups in CATIE and STAR*D. The interpretation of the other MDS dimensions was more ambiguous–they not strongly related to self-reported ethnicity, suggesting that they capture more subtle (cryptic) dimensions of population structure [33].

In Table 3 we show results from multiple regression analyses comparing the predictive power of our ancestral dimensions versus self-reported ethnicity. In order to compare models, we start with a full model #1 that includes all 5 MDS dimensions plus the 3 ethnicity variables. Model #2 includes the 5 MDS dimensions only. Compared to the full model #1, dropping the 3 Ethnicity variables decreased the correlations on average by 0.046. Even when controlling the FDR [30] at the 0.95 level, meaning that 95% of the significant results are expected to be false discoveries, this decrease was not significant for any of the 137 tested drug-outcome combinations. Conversely, dropping the 5 MDS dimensions reduced the correlations on average by 0.085 where for a number of tested drug outcome combinations revealed the decrease was significant at FDR levels of 0.5 and 0.95. Thus, these results suggest that the marginal explanatory power of the MDS dimensions was generally greater than self-reported ancestry. The final model # 4, including the 3 MDS and 3 ethnicity variables, was used to test whether the 2 MDS dimensions that did not correlated strongly with self-reported ethnicity (Supporting Information S1) did contribute to the predicting of drug response. Dropping these 2 MDS dimensions resulted in an average decrease in correlations of 0.030, with 3, 7, and 25 tests being significant at FDR levels of 0.1, 0.5, and 0.95. Thus, rather than being technical artifacts, these 2 MDS dimensions appeared to capture meaningful ancestral differences that did contribute to the prediction of drug response.

**Table 3:** Multiple regressions analyses predicting antipsychotic and antidepressant treatment response and side-effects using ancestral background.

Model		CATIE						STAR*D					
		Multiple correlation			# significant results			Multiple correlation			# significant results		
		Mean	1st Qu	3rd Qu	q<0.10	q<0.50	q<0.95	Mean	1st Qu	3rd Qu	q<0.10	q<0.50	q<0.95
1	5 MDS +3 Ethnicity	0.239	0.174	0.291	1	11	114	0.248	0.186	0.305	4	16	85
2	5 MDS	0.193	0.131	0.246	6	44	116	0.201	0.143	0.243	4	13	86
3	3 Ethnicity	0.154	0.091	0.187	2	40	120	0.158	0.101	0.203	10	26	81
4	3 MDS +3 Ethnicity	0.209	0.136	0.254	2	24	112	0.212	0.152	0.271	7	17	82
**Model comparison**		**Δ multiple correlations**			**Δ # significant results**			**Δ multiple correlations**			**Δ # significant results**		
		**Mean**	**1st Qu**	**3rd Qu**	**q<0.10**	**q<0.50**	**q<0.95**	**Mean**	**1st Qu**	**3rd Qu**	**q<0.10**	**q<0.50**	**q<0.95**
2 vs. 1	Drop 3 Ethnicity	−0.046	−0.061	−0.016	0	0	0	−0.047	−0.067	−0.017	0	8	13
3 vs. 1	Drop 5 MDS	−0.085	−0.114	−0.046	0	2	40	−0.089	−0.123	−0.046	0	0	72
4 vs. 1	Drop 2 MDS	−0.030	−0.043	−0.007	3	7	25	−0.036	−0.047	−0.009	0	0	87

MDS – multidimensional scaling. Qu – quartile.

In the model comparison section, reductions in the multiple correlations or number of significant results indicate that the compared model had less predictive power than model #1.

While the results presented in Table 3 describe a systematic trend of substantial marginal effects for the MDS dimensions, over and above the effects of self-reported ethnicity, they do not provide formal statistical tests of these marginal effects. Thus, in table 4 we present results from F-tests of model fit quantifying the statistical significance of the marginal effects of the unique MDS dimensions, over and above the effects of self-report ethnicity. These results show that for CATIE, 8.1% of models showed a significant (*p*<0.05) improvement to model fit with the inclusion of these MDS variables. A Chi-squared test of proportions indicated that this 0.081 proportion was significantly greater than the 0.05 proportion expected under the null (χ^2^ statistic = 2.731; χ^2^ *p*-value = 0.049). For STAR*D, results for the MDS dimensions were even stronger with 10.2% of models showing significant improvement to model fit with the inclusion of the MDS variables. A Chi-squared test of proportions indicated that this 0.102 proportion was significantly greater than the 0.05 expected under the null (χ^2^ statistic = 5.062; χ^2^ *p*-value = 0.012).

**Table 4:** F-tests of the marginal effects of unique MDS dimensions and self-reported ethnicity.

CATIE				
Model	# significant	# significant/all	khgr;^2^ statistic	khgr;^2^ *p*-value
MDS	11	0.081	2.731	0.049
Ethnicity	4	0.029	1.214	0.865
STAR*D				
**Model**	**# significant**	**# significant/all**	**χ^2^ statistic**	**χ^2^** ***p*** **-value**
MDS	9	0.102	5.062	0.012
Ethnicity	8	0.091	3.100	0.039

MDS – multidimensional scaling.

For both samples, in more models than expected by chance the marginal effect of the MDS dimensions significantly improved model fit, over and above self-reported ethnicity. Self-reported ethnicity showed mixed statistical evidence of improving model fit conditional on the MDS dimensions.

For comparison purposes, we applied the same approach to test the marginal effects of self-reported ethnicity, over and above the effects of the MDS ancestral dimensions. The proportions of significant marginal effects for self-reported ethnicity were smaller than those for the MDS dimensions in both samples. Chi-squared test of proportions for the self-reported ethnicity marginal effects showed mixed evidence, with no significant difference from the null expectation in CATIE (χ^2^ statistic = 1.214; χ^2^ *p*-value = 0.865) and a modestly significant result in STAR*D (χ^2^ statistic = 3.100; χ^2^ *p*-value = 0.039). In sum, these results demonstrate that the MDS dimensions explained significant amounts of outcome variance, over and above that explained by self-reported ethnicity, in both samples. Further, there is some modest evidence that self-reported ethnicity may also provide some unique predictive power, beyond that capture by genotype-based ancestry, for antidepressant drug response.

Finally, as proof of concept that genotype-based ancestry could potentially be applied in clinical settings, for each of the 5 ancestry dimension, we identified 700 SNPs jointly capturing approximately the same information as the genome-wide MDS measures. To do this we proceeded via the following steps. First, we pruned the genotype data to include only markers in linkage equilibrium (pairwise R^2^<0.1) using PLINK’s “indep” function [15]. Next, for each dimension, we sorted the absolute MDS loadings for the pruned genotype data and selected 700 SNPs with the strongest loadings. Finally, while the loadings themselves could be used as weights to calculate proxy MDS scores, to optimize performance of the scores we regressed each MDS dimension on its 700 top SNPs (using a single multiple regression model) and used the resulting coefficients as weights in calculating proxy MDS scores. For all samples and MDS dimensions, the 700 SNP proxy model explained >99% of variance in the MDS dimension. Thus, the proxy dimensions were virtually identical to genome-wide MDS dimensions, and accordingly, provided equivalent results when substituted in the primary analysis. In Supporting Information S2, we provide these empirically determined SNP lists and the weights used to calculate proxy dimensions as a resource for researchers interested in replicating these findings or extending efforts toward developing predictive algorithms of psychiatric drug response.

## Discussion

Genome-wide estimates of each patient’s unique mosaic of ancestral backgrounds mediated the effects of all studied antipsychotic and antidepressant drugs on a wide range of efficacy and toxicity outcomes. This evidence is convincing not only as a result of the remarkably pervasive associations seen in Figure 1, but also because of the quality and size of the psychiatric clinical trial samples analyzed.

One potential explanation of why these effects are so pervasive is that ancestral differences typically involve a large number of genetic variants. For instance, over 400,000 markers were significantly associated with the first MDS dimension in the CATIE sample. Because the allele frequencies of so many variants contribute to each ancestral dimension, there are likely to be different subsets of variants that are relevant to response for any given drug. According to this logic, even though the specific variants comprising the relevant subset for any given drug-outcome combination may be unknown, the pervasive genetic differences captured by the ancestral dimensions are still likely to have general prognostic value in predicting treatment response. Consequently, it can be expected that the proposed method will generalize to a wide variety of other drug-treatment response combinations.

Our results provide compelling evidence that ancestral information powerfully predicts a range of antidepressant and antipsychotic treatment outcomes. However, establishing that ancestry influences drug response gives rise to the question: Is there added value in genotype-based ancestral dimensions over and above that provided by self-reported ethnicity, which is less expensive and easier to measure. The results of the current study suggest that, yes–there is indeed additional value in genotype-based ancestry. As shown in Table 4, the marginal effects of unique genotype-based ancestral dimensions provide significant predictive power over and above self-report ancestry in both the CATIE and STAR*D studies.

There are several likely reasons for this phenomenon. First, genetic ancestry is a mosaic of many dimensions that cannot be captured with a discrete variable comprising few categories. Indeed, our analyses indicated that while some MDS dimensions corresponded to self-reported ethnicity, others appeared to capture population differences not measured in conventional race/ethnicity questionnaires. In addition to being more nuanced and exhaustive measures of ancestral background, there are statistical advantages of using quantitative ancestral dimensions. First, the reduction of statistical power when using categorical versus quantitative variables is a well-established phenomenon [34], [35]. For example, dichotomizing a predictor at its median reduces variance explained in a normally distributed outcome by 38%, with further reductions as the dichotomization point moves away from the median [34]. Another advantage of using a quantitative measure of ancestry in the prediction algorithm is the possibility to study the full ancestry spectrum leading to an extension of personalized medicine to groups that are less represented or have been historically understudied. For example, due to low sample sizes, minority racial/ethnic groups are often dropped from analyses to avoid estimation problems. However, by assessing their unique ancestral make-up using quantitative dimensions, they can more readily be included in analyses. None of this is to say, however, that self-reported ethnicity is without value in the study of drug response. While the predictive power of self-reported ethnicity did not match that of genotype-based ancestral dimensions; as shown in row 3 of Table 3, self-reported ethnicity-only models did show nontrivial prognostic power in predicting psychiatric drug response. Further, we observed some tentative evidence (in STAR*D, but not CATIE) that self-reported ethnicity has predictive power over and above genotype-based ancestry. While the tentative nature of this evidence suggests the need for further study before drawing strong conclusions, if replicated, this result would be consistent with social constructionist theories of race arguing that the social categorization of race/ethnicity has a medical significance independent of genetic differences [36], [37], [38]. Such a conclusion would be unsurprising in light of existing research suggesting ethnically-mediated social effects on psychiatric treatment response, such as variation in adherence to antidepressant treatment by English proficiency between and within ethnic groups [39]. In sum, social categories measured by self-reported ethnicity may yield additional information to genetic ancestry, due to capturing social constructed influences that do not correspond precisely to genetic ancestry.

While predictive algorithms to personalize psychiatric treatment are still relatively early in development, this research provides proof of principle that genotype-based ancestry could easily be incorporated into such future clinical applications. In this scenario, after collecting genotype data, a pre-existing algorithm could be used to estimate the ancestral mosaic of new patients. Ideally, this pre-existing algorithm would be derived from a large, geographically diverse sample of subjects. Such a sample would avoid the issue of certain ancestral dimensions remaining undetected, which would result in reduced predictive power. Cost of genotyping should not present an obstacle. Although we had access to over 400,000 polymorphisms, as we empirically demonstrate above, proxy ancestral dimensions can be calculated using far fewer genetic markers. Thus, ancestral dimension scores could be generated using low-end genotyping arrays currently available for tens of dollars for use in clinical settings. Clearly, the current effort is only an initial step towards individualizing treatment and we envision various ways in which this method may be modified to further increase predictive power. One shortcoming of the method used here to select and combine markers into proxy ancestral dimensions is its susceptibility to overfitting. Thus, future efforts may consider machine learning methods that explicitly account for overfitting [40], [41]. Further, instead of basing SNP selection on agnostic genome-wide analyses, the choice of markers could be tailored to the specific population being studied to obtain more refined ancestral measures. Such an extension could draw on existing knowledge of ancestry informative markers (AIMs) [33], [42], [43], [44], which are specific panels of markers selected to optimally assess ancestral differences.

The proposed method relies on the premise that a variable need only be robustly associated with drug response to constitute an effective predictor. This is clearly inferior to the use of causal genetic markers that would provide a biological rationale and facilitate further insight into the pathological process. However, finding, replicating and validating causal markers predicting response to a wide variety of drug-indication combinations is apt to remain a challenging and slow-progressing process for the foreseeable future. Reasons for this include, for instance, the fact that clinical trials are often unique, with modest sample sizes due to the enormous cost of conducting these studies. This makes it difficult to replicate findings in independent samples or to detect markers with small effects due to insufficient statistical power. In the meantime, the proposed method may serve as a potent, immediate starting point for developing algorithms predicting the most effective and least toxic medication for a wide variety of drug-indication combinations. As relatively few new psychiatric drugs are currently under development, such personalized medicine offers a promising, currently feasible approach toward optimizing pharmacotherapy for psychiatric conditions.

## Supporting Information

Supporting Information S1 Technical information describing study design, data structure, genotyping, treatment effect and MDS estimation, and associations between MDS ancestral dimension and self-reported ethnicity for both CATIE AND STAR*D.
(DOC)

Supporting Information S2 Empirically determined SNP lists and weights used to calculate proxy MDS ancestral dimensions using 700 SNP subsets of the CATIE and STAR*D genomewide data.
(XLSX)

## References

[1] Ashley EA, Butte AJ, Wheeler MT, Chen R, Klein TE, et al (2010) Clinical assessment incorporating a personal genome. Lancet 375: 1525–1535. 20435227 10.1016/S0140-6736(10)60452-7 PMC2937184

[2] Simon GE, Von Korff M, Rutter CM, Peterson DA (2001) Treatment process and outcomes for managed care patients receiving new antidepressant prescriptions from psychiatrists and primary care physicians. Arch Gen Psychiatry 58: 395–401. 11296101 10.1001/archpsyc.58.4.395

[3] Couzin J (2008) Science and commerce. Gene tests for psychiatric risk polarize researchers. Science 319: 274–277. 18202268 10.1126/science.319.5861.274

[4] Braff DL, Freedman R (2008) Clinically responsible genetic testing in neuropsychiatric patients: a bridge too far and too soon. Am J Psychiatry 165: 952–955. 18676598 10.1176/appi.ajp.2008.08050717

[5] Crowley JJ, Sullivan PF, McLeod HL (2009) Pharmacogenomic genome-wide association studies: lessons learned thus far. Pharmacogenomics 10: 161–163. 19207016 10.2217/14622416.10.2.161 PMC3893087

[6] Holden C (2003) Race and medicine. Science 302: 594–596. 14576420 10.1126/science.302.5645.594

[7] Wilson JF, Weale ME, Smith AC, Gratrix F, Fletcher B, et al (2001) Population genetic structure of variable drug response. Nat Genet 29: 265–269. 11685208 10.1038/ng761

[8] Clark SL, Adkins DE, van den Oord E (2011) Analysis of efficacy and side effects in CATIE demonstrates drug response subgroups and potential for personalized medicine. Schizophrenia Research 132: 114–120. 21872442 10.1016/j.schres.2011.07.031 PMC3195895

[9] Lieberman JA, Stroup TS, McEvoy JP, Swartz MS, Rosenheck RA, et al (2005) Effectiveness of antipsychotic drugs in patients with chronic schizophrenia. N Engl J Med 353: 1209–1223. 16172203 10.1056/NEJMoa051688

[10] Trivedi MH, Rush AJ, Wisniewski SR, Nierenberg AA, Warden D, et al (2006) Evaluation of outcomes with citalopram for depression using measurement-based care in STAR*D: implications for clinical practice. Am J Psychiatry 163: 28–40. 16390886 10.1176/appi.ajp.163.1.28

[11] Stroup TS, McEvoy JP, Swartz MS, Byerly MJ, Glick ID, et al (2003) The National Institute of Mental Health Clinical Antipsychotic Trials of Intervention Effectiveness (CATIE) project: schizophrenia trial design and protocol development. Schizophr Bull 29: 15–31. 12908658 10.1093/oxfordjournals.schbul.a006986

[12] Rush AJ, Fava M, Wisniewski SR, Lavori PW, Trivedi MH, et al (2004) Sequenced treatment alternatives to relieve depression (STAR*D): rationale and design. Control Clin Trials 25: 119–142. 15061154 10.1016/s0197-2456(03)00112-0

[13] Sullivan PF, Lin D, Tzeng JY, van den Oord E, Perkins D, et al (2008) Genomewide association for schizophrenia in the CATIE study: results of stage 1. Mol Psychiatry 13: 570–584. 18347602 10.1038/mp.2008.25 PMC3910086

[14] Garriock HA, Kraft JB, Shyn SI, Peters EJ, Yokoyama JS, et al (2010) A Genomewide Association Study of Citalopram Response in Major Depressive Disorder. Biological Psychiatry 67: 133–138. 19846067 10.1016/j.biopsych.2009.08.029 PMC2794921

[15] Purcell S, Neale B, Todd-Brown K, Thomas L, Ferreira MA, et al (2007) PLINK: a tool set for whole-genome association and population-based linkage analyses. Am J Hum Genet 81: 559–575. 17701901 10.1086/519795 PMC1950838

[16] Price AL, Patterson NJ, Plenge RM, Weinblatt ME, Shadick NA, et al (2006) Principal components analysis corrects for stratification in genome-wide association studies. Nat Genet 38: 904–909. 16862161 10.1038/ng1847

[17] McClay JL, Adkins DE, Aberg K, Bukszar J, Khachane AN, et al (2011) Genome-wide pharmacogenomic study of neurocognition as an indicator of antipsychotic treatment response in schizophrenia. Neuropsychopharmacology 36: 616–626. 21107309 10.1038/npp.2010.193 PMC3055694

[18] Aberg K, Adkins DE, Liu Y, McClay JL, Bukszar J, et al.. (2010) Genome-wide association study of antipsychotic-induced QTc interval prolongation. Pharmacogenomics J. 10.1038/tpj.2010.76 PMC3388904 20921969

[19] Adkins DE, Aberg K, McClay JL, Hettema JM, Kornstein SG, et al (2010) A genomewide association study of citalopram response in major depressive disorder-a psychometric approach. Biol Psychiatry 68: e25–27. 20619826 10.1016/j.biopsych.2010.05.018 PMC2929324

[20] Adkins DE, Aberg K, McClay JL, Bukszar J, Zhao Z, et al (2011) Genomewide pharmacogenomic study of metabolic side effects to antipsychotic drugs. Mol Psychiatry 16: 321–332. 20195266 10.1038/mp.2010.14 PMC2891163

[21] Aberg K, Adkins DE, Bukszar J, Webb BT, Caroff SN, et al (2010) Genomewide association study of movement-related adverse antipsychotic effects. Biol Psychiatry 67: 279–282. 19875103 10.1016/j.biopsych.2009.08.036 PMC3388725

[22] McClay JL, Adkins DE, Aberg K, Stroup S, Perkins DO, et al (2011) Genome-wide pharmacogenomic analysis of response to treatment with antipsychotics. Mol Psychiatry 16: 76–85. 19721433 10.1038/mp.2009.89 PMC2888895

[23] Adkins DE, Clark SL, Aberg K, Hettema JM, Bukszar J, et al (2012) Genome-wide pharmacogenomic study of citalopram-induced side effects in STAR*D. Translational Psychiatry 2: e129. 22760553 10.1038/tp.2012.57 PMC3410623

[24] Adkins DE, Khachane AN, McClay JL, Aberg K, Bukszar J, et al (2012) SNP-based analysis of neuroactive ligand-receptor interaction pathways implicates PGE2 as a novel mediator of antipsychotic treatment response: Data from the CATIE study. Schizophrenia Research 135: 200–201. 22099390 10.1016/j.schres.2011.11.002 PMC3515657

[25] Garriock HA, Kraft JB, Shyn SI, Peters EJ, Yokoyama JS, et al (2010) A genomewide association study of citalopram response in major depressive disorder. Biol Psychiatry 67: 133–138. 19846067 10.1016/j.biopsych.2009.08.029 PMC2794921

[26] Anderson CA, Pettersson FH, Clarke GM, Cardon LR, Morris AP, et al (2010) Data quality control in genetic case-control association studies. Nat Protoc 5: 1564–1573. 21085122 10.1038/nprot.2010.116 PMC3025522

[27] van den Oord EJ, Adkins DE, McClay J, Lieberman J, Sullivan PF (2009) A systematic method for estimating individual responses to treatment with antipsychotics in CATIE. Schizophr Res 107: 13–21. 18930379 10.1016/j.schres.2008.09.009 PMC2652489

[28] Robinson GK (1991) That BLUP is a Good Thing: The Estimation of Random Effects. Statistical Science 6: 15–32.

[29] Clark SL, Adkins DE, Aberg K, Hettema JM, McClay JL, et al (2012) Pharmacogenomic study of side-effects for antidepressant treatment options in STAR*D. Psychological Medicine 42: 1151–1162. 22041458 10.1017/S003329171100239X PMC3627503

[30] Benjamini Y, Hochberg Y (1995) Controlling the False Discovery Rate - a Practical and Powerful Approach to Multiple Testing. Journal of the Royal Statistical Society Series B-Methodological 57: 289–300.

[31] Storey J (2003) The positive false discovery rate: A Bayesian interpretation and the q-value. Ann Stat 31: 2013–2035.

[32] de Bakker PIW, Ferreira MAR, Jia XM, Neale BM, Raychaudhuri S, et al (2008) Practical aspects of imputation-driven meta-analysis of genome-wide association studies. Human Molecular Genetics 17: R122–R128. 18852200 10.1093/hmg/ddn288 PMC2782358

[33] Price AL, Butler J, Patterson N, Capelli C, Pascali VL, et al (2008) Discerning the ancestry of European Americans in genetic association studies. PLoS Genet 4: e236. 18208327 10.1371/journal.pgen.0030236 PMC2211542

[34] Cohen J (1983) The Cost of Dichotomization. Applied Psychological Measurement 7: 249–253.

[35] MacCallum RC, Zhang SB, Preacher KJ, Rucker DD (2002) On the practice of dichotomization of quantitative variables. Psychological Methods 7: 19–40. 11928888 10.1037/1082-989x.7.1.19

[36] Mountain JL, Risch N (2004) Assessing genetic contributions to phenotypic differences among ‘racial’ and ‘ethnic’ groups. Nature Genetics 36: S48–S53. 15508003 10.1038/ng1456

[37] Karter AJ (2003) Commentary: Race, genetics, and disease– in search of a middle ground. International Journal of Epidemiology 32: 26–28. 12690000 10.1093/ije/dyg033

[38] Williams DR, Sternthal M (2010) Understanding Racial-ethnic Disparities in Health: Sociological Contributions. Journal of Health and Social Behavior 51: S15–S27. 20943580 10.1177/0022146510383838 PMC3468327

[39] Olfson M, Marcus SC (2009) National patterns in antidepressant medication treatment. Arch Gen Psychiatry 66: 848–856. 19652124 10.1001/archgenpsychiatry.2009.81

[40] Zou H, Hastie T, Tibshirani R (2006) Sparse principal component analysis. Journal of Computational and Graphical Statistics 15: 265–286.

[41] Paul D, Bair E, Hastie T, Tibshirani R (2008) “Preconditioning” for feature selection and regression in high-dimensional problems’. Annals of Statistics 36: 1595–1618.

[42] Tian C, Gregersen PK, Seldin MF (2008) Accounting for ancestry: population substructure and genome-wide association studies. Hum Mol Genet 17: R143–150. 18852203 10.1093/hmg/ddn268 PMC2782357

[43] Tian C, Hinds DA, Shigeta R, Kittles R, Ballinger DG, et al (2006) A genomewide single-nucleotide-polymorphism panel with high ancestry information for African American admixture mapping. Am J Hum Genet 79: 640–649. 16960800 10.1086/507954 PMC1592561

[44] Tian C, Hinds DA, Shigeta R, Adler SG, Lee A, et al (2007) A genomewide single-nucleotide-polymorphism panel for Mexican American admixture mapping. Am J Hum Genet 80: 1014–1023. 17557415 10.1086/513522 PMC1867091

