# Supplementary material for: Genotype-Based Ancestral Background Consistently Predicts Efficacy and Side Effects across Treatments in CATIE and STAR*D
Source: PLoS One. 2013 Feb 6;8(2):e55239. doi: 10.1371/journal.pone.0055239 (PMC3566192; doi:10.1371/journal.pone.0055239)
Supplement: Supporting Information S1 — Technical information describing study design, data structure, genotyping, treatment effect and MDS estimation, and associations between MDS ancestral dimension and self-reported ethnicity for both CATIE AND STAR*D. (DOC) [file pone.0055239.s001.doc]

**Supporting Information S1 for the paper:**

**“Genotype-based ancestral background consistently predicts efficacy and side effects across treatments in CATIE and STAR*D”**

1. **Antipsychotic Trial of Intervention Effectiveness (CATIE)**

Subjects and study design Page 2

Assessment instruments Page 2

Estimating treatment effects Page 4

Genotyping Page 5

Ancestral dimensions Page 5

1. **The Sequenced treatment alternatives to relieve depression (STAR*D) study**

Subjects and study design Page 7

Assessment instruments Page 7

Estimating treatment effects Page 7

Genotyping Page 7

Ancestral dimensions Page 8

1. **Correlations between self-reported ethnicity and MDS ancestral dimensions, both studies**

Table S1: Correlation matrix Page 9

1. **Antipsychotic Trial of Intervention Effectiveness (CATIE).**

Subjects and study design

This study includes the subjects (N=765) from the Clinical Antipsychotic Trial of Intervention Effectiveness (CATIE). This study sample has been carefully described elsewhere ^1, 2^. In short, CATIE is a multiphase randomized controlled trial of six antipsychotic medications, including clozapine, olanzapine, perphenazine, quetiapine, risperidone and ziprasidone, which followed patients for up to 18 months. To maximize representativeness, the participants were recruited from 57 clinical settings around the United States. Patients were diagnosed with schizophrenia using the Structured Clinical Interview for DSM-IV ^3^. Their mean age was 40.9 years and on average they first received antipsychotic medication 14.3 years previously. All patients or their legal guardians gave written informed consent, including consent for genetic studies, and the institutional review board at each site approved the study.

Assessment instruments

*Efficacy*: The Positive and Negative Syndrome Scale (PANSS) ^4^ is used to assess improvement of schizophrenia symptoms. The 30 items of the PANSS measure a broad range of the symptoms typical for schizophrenia where five factors are generally preferred to represent its underlying structure^5, 6^. More specifically, because of their large sample size (N = 5,769), we used the five scales derived by Van der Gaag et al. ^7^ labeled Positive symptoms, Negative symptoms, Disorganization symptoms, Excitement, and Emotional distress.

Neurocognition is moderately to severely impaired in patients with schizophrenia. For CATIE, a neurocognitive advisory group consisting of numerous leaders in the field was convened and arrived at consensus regarding the contents, standardization, and methodology of the neurocognitive assessment battery ^8, 9^. Eleven neurocognitive tests were administered, resulting in 24 individual scores. Factor analysis showed that a model comprised of five domain scores provided the best fit^9^. The five neurocognitive domains were: Processing Speed that was based on the standardized mean of Grooved Pegboard^10^, WAIS-R digit Symbol Test^11^, and the mean of the two verbal fluency measures^12^. Verbal Memory was assessed with the Hopkins Verbal Learning Test (average of 3 trials)^13^. Vigilance was based on the Continuous Performance Test d-prime scores (average of 2-digit, 3-digit, and 4-digit)^14^. Reasoning Summary Score was the mean of Wisconsin Card Sorting Test^15^ and WISC-R Mazes^16^. Working Memory Summary Score was the average of a computerized test of visuospatial working memory (sign reversed) ^17^ and letter number sequencing ^18^. The correlations among the five domain scores were medium to high and we therefore also analyze the composite score^9^ of these five domains.

*Side effects*: Antipsychotic treatments have been associated with a variety of metabolic side effects and high blood pressure^19-21^. The medical consequences range from cosmetic issues to increased rates of cardiovascular disease (e.g., hypertension, coronary artery disease) and diabetes^22^. The metabolic measures collected in CATIE have been described in detail previously^23, 24^. To briefly restate, BMI (kg/m^2^) was calculated in the standard fashion and waist and hip circumferences (inches) were measured at the narrowest and widest points, respectively. Blood pressure (mm Hg) was measured as a single, seated determination, and heart rate (bpm) was measured as resting pulse count in 30 seconds×2. The lipd panel included triglycerides (mg/dL), total cholesterol (mg/dL) and HDL(mg/dL)). In addition to fasting blood glucose levels (mg/dL), we studied hemoglobin A1C (%) that is often used as a biomarker to determine the average [plasma](http://en.wikipedia.org/wiki/Blood_plasma) [glucose](http://en.wikipedia.org/wiki/Glucose) [concentration](http://en.wikipedia.org/wiki/Concentration) over longer periods of time. All metabolic laboratory measures were assessed at a single central laboratory. For assessment of laboratory measures, CATIE subjects were asked to present in a fasting state. However, as information on last meal was collected and suggested a significant range; we explicitly adjusted glucose and triglycerides for fasting time using a regression-based approach.

Antipsychotics are among the drugs causing QT prolongation, an electrocardiogram (ECG) measure associated with an increased risk of cardiac arrhythmias and sudden death ^25-28^. Although sudden death are rare, the severity of this event makes QT prolongation on of the most common reasons pharmaceuticals are restricted or removed from the market. Prior to analyses, the QT interval was corrected for the heart rate (QTc) according to Bazett’s method ^29^.

Estimating treatment effects

To estimate treatment effects in CATIE, we developed a systematic method ^30^. Using mixed modeling ^31, 32^ our method first determines the optimal functional form of over-time drug response, then screens many possible covariates to select those that improve the precision of the treatment effect estimates, and finally generates the individual treatment effect estimates based on the best fitting model using best linear unbiased predictors (BLUPs) ^33^. As our approach condenses all information collected during the trials in an optimal, empirical fashion, it results in more precise estimates than traditional approaches (e.g., subtracting pre- from post-treatment observations) that estimate treatment effects using only two assessments.

Specifically, to determine the optimal drug response trajectory for each outcome we fit a series of models specifying linear change for a given number of days on drug and flat thereafter. This series began with a model assuming that maximal drug response was achieved at day one. Each subsequent model specified an incrementally longer duration until maximal drug response was achieved with the final model assuming that the drug effect did not plateau (i.e. linear change throughout the trial). The model with the best fit of series was selected to determine the average number of days until maximal drug response. After determining the optimal functional form of the drug response trajectories, 36 covariates were screened to identify those that improved the precision of the treatment effect estimates. These covariates consisted of design characteristics, socio-demographic measures, clinical information, confounding medications, and baseline antipsychotic treatment. The number of selected covariates ranged from zero (e.g. for BMI, waist and hip circumference) to four or greater (e.g. blood lipids). Design characteristics where among the most commonly selected covariates. Finally, treatment effects were generated by employing a unique feature of the mixed model—*random effects*. To elaborate, the mixed model estimates two types of parameters, coefficients that describe the predictors’ average effects for the full sample (i.e., fixed effects) and deviations from the average effects for each subject (i.e., random effects). Thus, for each of the six trial drugs investigated, we were able to output treatment effects as random drug effects. Intuitively, these treatment effects quantify how much, for instance, each subject’s metabolic phenotypes change in response to a given drug, relative to the average effect for all subjects who took the drug. Out of all possible treatment effect measures, in 10 instances there were no significant individual differences in drug response. These treatment effects were omitted from further analyses. This left of total of 137 drug-outcome combinations.

Genotyping

DNA sampling, genotyping and genotype quality control have been described by Sullivan et al.^34^. In total, 665,439 SNPs were genotyped using the Affymetrix 500K chipset (Santa Clara, CA, USA) and a custom 164K chip created by Perlegen (Mountain View, CA, USA). After quality control, genotypes for 492,900 SNPs from 738 individuals remained for investigating ancestral background dimensions.

Ancestral dimensions

To estimate the ancestral dimensions we used the MDS approach as implemented in PLINK ^40^. Input data for this approach were the genomewide average proportion of alleles shared identical by state (IBS) between any two individuals. The first ancestral dimension from this genetic similarity matrix captures the maximal variance in the genetic similarity; the second dimension must be orthogonal to the first and captures the maximum amount of residual genetic similarity; and so on. We specified in PLINK the extraction of 8 MDS dimensions. Next, we performed genome-wide association analyses with each of these 8 dimensions as outcome variables and counted for each dimension the number of significant results using an FDR threshold of 0.1.

Figure 1. Number of significant dimension-SNP associations after controlling the FDR at the 0.1 level.

Figure 1 shows that over 400K SNPs were significant associated with the first dimension. This indicated that this dimension was associated with allele frequency differences for the vast majority of SNPs. A substantial number of SNPs has allele frequency differences for dimensions 1-5. However, the number of SNPs associated with the dimensions 6-8 remained fairly constant and did not seems to capture substantial genetic similarity in the sample anymore. Hence, we chose to use the first 5 dimensions for further analyses.

1. **The Sequenced treatment alternatives to relieve depression (STAR*D) study**

Subjects and study design

The Sequenced treatment alternatives to relieve depression (STAR*D) study is a prospective, randomized clinical trial of outpatients with nonpsychotic major depressive disorder (MDD) ^41^. Sample collection involved 41 clinical sites across the US. The full clinical trial study sample includes 4,000 adults (ages 18–75) from both primary and specialty care practices who had shown neither inadequate response or intolerance to any of the protocol treatments. The study consisted of 4 phases. In the first phase all patients started with citalopram, a selective serotonin reuptake inhibitor antidepressant. Different medications or medication combinations for treatment resistant subjects were administered in each subsequent phase.

Assessment instruments

The outcomes examined here involved clinician and self reports of depression symptoms using the Quick Inventory of Depressive Symptomatology (QIDS) ^42^. The QIDS assesses all the diagnostic MDD symptom domains designated by the 4th edition of the American Psychiatry Association Diagnostic and Statistical Manual of Mental Disorders (DSM-IV ^43^ and is frequently used for measuring of symptom severity. QIDS assessments were obtained frequently during the course of the trial with the total number of assessments for the entire sample exceeding 14,000 for both clinician and self ratings. The clinician and self ratings correlated highly (*r* = .87) in STAR*D suggesting good inter-rater reliability.

Estimating treatment effects

To estimate the treatment effects we used the same methods as described above for the CATIE sample.

Genotyping

NIMH funded genomewide genotyping for a subsample of patients using Affymetrix 500K arrays. Genotyping of the STAR*D samples was conducted at two locations and on two different platforms. A total of 969 subjects were genotyped at Affymetrix, Inc. (South San Francisco) on the Human Mapping 500K Array Set. We genotyped the remaining 979 samples using the Affymetrix Genome-Wide Human SNP Array 5.0. The two groups were balanced by ethnic grouping, gender and proportions of responders and non-responders. Samples run on Affymetrix 500K Array were called using the BRLMM algorithm, and samples analyzed on Affymetrix Array 5.0 were called using the BRLMM-P algorithm. Twelve samples were genotyped on both the 500K and 5.0 Arrays, and we found > 99% concordance across these platforms.

Ancestral dimensions

MDS dimensions and outcomes (self and clinician rated efficacy as measured by the QIDS) were constructed using the same approaches as used in CATIE (see above). As with CATIE, five MDS dimensions were selected for STAR*D. We focused on drugs and drug combinations for which there were at least 100 subjects.

1. **Correlations between self-reported ethnicity and MDS ancestral dimensions, both studies**

**Table S1: Correlation matrix of self-reported ethnicity to ancestral MDS**

|  | STAR*D |  |  | CATIE |  |  |
| --- | --- | --- | --- | --- | --- | --- |
|  | White | Black | Hispanic | White | Black | Hispanic |
| MDS1 | -0.016 | -0.008 | 0.036 | 0.938 | -0.969 | 0.098 |
| MDS2 | 0.817 | -0.902 | 0.037 | -0.090 | -0.112 | 0.546 |
| MDS3 | -0.026 | 0.120 | -0.743 | 0.214 | -0.016 | 0.569 |
| MDS4 | 0.003 | -0.005 | -0.003 | -0.014 | 0.012 | 0.001 |
| MDS5 | -0.018 | 0.041 | -0.009 | 0.008 | -0.008 | -0.001 |

Reference List

1. Lieberman JA, Stroup TS, Mcevoy JP et al. Effectiveness of antipsychotic drugs in patients with chronic schizophrenia. N Engl J Med 2005; 353(12):1209-1223.

2. Stroup TS, McEvoy JP, Swartz MS et al. The National Institute of Mental Health Clinical Antipsychotic Trials of Intervention Effectiveness (CATIE) project: schizophrenia trial design and protocol development. Schizophr Bull 2003; 29(1):15-31.

3. First M, Spitzer R, Gibbon M, Williams J. Structured Clinical Interview for DSM-IV Axis I Disorders--Administration Booklet. Washington D.C.: American Psychiatric Press, Inc.; 1994.

4. Kay SR, Fiszbein A, Opler LA. The positive and negative syndrome scale (PANSS) for schizophrenia. Schizophr Bull 1987; 13(2):261-276.

5. White L, Harvey PD, Opler L, Lindenmayer JP. Empirical assessment of the factorial structure of clinical symptoms in schizophrenia. A multisite, multimodel evaluation of the factorial structure of the Positive and Negative Syndrome Scale. The PANSS Study Group. Psychopathology 1997; 30(5):263-274.

6. Van den Oord EJCG, Rujescu D, Robles JR et al. Factor structure and external validity of the PANSS revisited. Schizophrenia Research 2006; 82(2-3):213-223.

7. van der Gaag M, Hoffman T, Remijsen M et al. The five-factor model of the Positive and Negative Syndrome Scale II: a ten-fold cross-validation of a revised model. Schizophr Res 2006; 85(1-3):280-287.

8. Keefe RSE, Mohs RC, Bilder RM et al. Neurocognitive assessment in the Clinical Antipsychotic Trials of Intervention Effectiveness (CATIE) project schizophrenia trial: Development, methodology, and rationale. Schizophrenia Bulletin 2003; 29(1):45-55.

9. Keefe RS, Bilder RM, Harvey PD et al. Baseline Neurocognitive Deficits in the CATIE Schizophrenia Trial. Neuropsychopharmacology 2006.

10. Lafayette Instrument Company. Grooved Pegboard Instruction Manual (Model 32025). Lafayette, In: Lafayette Instrument Company; 1989.

11. Wechsler D. WAIS-R Manual: Wechsler Adult Intelligence Scale-Revised. New York: Psychological Corportation; 1981.

12. Benton AL, Hamscher K. Multilingual Aphasia Examination Manual (revised). Iowa City, IA: University of Iowa; 1978.

13. Brandt J. Wechsler Adult Intelligence Scale-Revised l memory test with six equivalent forms. The Clinical Neuropsychologist 1991; 5:125-142.

14. Cornblatt BA, Risch NJ, Faris G, Friedman D, Erlenmeyerkimling L. The Continuous Performance-Test, Identical Pairs Version (Cpt-Ip) .1. New Findings About Sustained Attention in Normal-Families. Psychiatry Research 1988; 26(2):223-238.

15. Heaton RK, Chelune GJ, Taley JL, Kay GG, Curtiss G. Wisconsin Card Sorting Test Manual: Revised and Expanded. Odessa, FL: Psychological Assessment Resources; 1993.

16. Wechsler D. Wechsler Intelligence Scale for Children. Third Edition ed. San Antonio, TX: Psychological Corportation; 1991.

17. Hershey T, Selke G, Fucetola R, Newcomner J.W. Spatial long-term memory but not working memory decreases over time in schizophrenia. Society for Neuroscience Abstracts 25, 572. 2005.

Ref Type: Abstract

18. Gold JM, Carpenter C, Randolph C, Goldberg TE, Weinberger DR. Auditory working memory and Wisconsin Card Sorting Test performance in schizophrenia. Archives of General Psychiatry 1997; 54(2):159-165.

19. Henderson DC. Weight gain with atypical antipsychotics: evidence and insights. J Clin Psychiatry 2007; 68 Suppl 12:18-26.

20. Allison DB, Mentore JL, Heo M et al. Antipsychotic-induced weight gain: a comprehensive research synthesis. Am J Psychiatry 1999; 156(11):1686-1696.

21. Henderson DC. Diabetes mellitus and other metabolic disturbances induced by atypical antipsychotic agents. Curr Diab Rep 2002; 2(2):135-140.

22. Henderson DC, Doraiswamy PM. Prolactin-related and metabolic adverse effects of atypical antipsychotic agents. J Clin Psychiatry 2008; 69 Suppl 1:32-44.

23. Mcevoy JP, Meyer JM, Goff DC et al. Prevalence of the metabolic syndrome in patients with schizophrenia: baseline results from the Clinical Antipsychotic Trials of Intervention Effectiveness (CATIE) schizophrenia trial and comparison with national estimates from NHANES III. Schizophr Res 2005; 80(1):19-32.

24. Meyer JM, Davis VG, Goff DC et al. Change in metabolic syndrome parameters with antipsychotic treatment in the CATIE Schizophrenia Trial: prospective data from phase 1. Schizophr Res 2008; 101(1-3):273-286.

25. Ray WA, Meredith S, Thapa PB, Meador KG, Hall K, Murray KT. Antipsychotics and the risk of sudden cardiac death. Arch Gen Psychiatry 2001; 58(12):1161-1167.

26. Stollberger C, Huber JO, Finsterer J. Antipsychotic drugs and QT prolongation. Int Clin Psychopharmacol 2005; 20(5):243-251.

27. Zareba W, Lin DA. Antipsychotic drugs and QT interval prolongation. Psychiatr Q 2003; 74(3):291-306.

28. Welch R, Chue P. Antipsychotic agents and QT changes. J Psychiatry Neurosci 2000; 25(2):154-160.

29. Bazett H. An analysis of the time-relations of electrocardiograms. Heart 1920; 7:353-370.

30. Van den Oord EJCG, Adkins DE, McClay J, Lieberman J, Sullivan PF. A systematic method for estimating individual responses to treatment with antipsychotics in CATIE. Schizophr Res 2009; 107:13-21.

31. Goldstein H. *Multilevel statistical models*. London: Arnold; 1995.

32. Searle SR, Casella G, McCulloch CE. Variance components. New York: Wiley; 1992.

33. Pinheiro JC, Bates DM. Mixed-effects models in S and S-plus. New York, NY: Springer.; 2000.

34. Sullivan PF, Lin D, Tzeng JY et al. Genomewide association for schizophrenia in the CATIE study: results of stage 1. Mol Psychiatry 2008; 13(6):570-584.

35. Devlin B, Roeder K. Genomic control for association studies. Biometrics 1999; 55(4):997-1004.

36. Pritchard JK, Stephens M, Rosenberg NA, Donnelly P. Association mapping in structured populations. Am J Hum Genet 2000; 67(1):170-181.

37. Price AL, Patterson NJ, Plenge RM, Weinblatt ME, Shadick NA, Reich D. Principal components analysis corrects for stratification in genome-wide association studies. Nat Genet 2006; 38(8):904-909.

38. Purcell S, Neale B, Todd-Brown K et al. PLINK: a tool set for whole-genome association and population-based linkage analyses. Am J Hum Genet 2007; 81(3):559-575.

39. Epstein MP, Allen AS, Satten GA. A simple and improved correction for population stratification in case-control studies. Am J Hum Genet 2007; 80(5):921-930.

40. Purcell S, Neale B, Todd-Brown K et al. PLINK: A Tool Set for Whole-Genome Association and Population-Based Linkage Analyses. Am J Hum Genet 2007; 81(3):559-575.

41. Rush AJ, Fava M, Wisniewski SR et al. Sequenced treatment alternatives to relieve depression (STAR*D): rationale and design. Control Clin Trials 2004; 25(1):119-142.

42. Rush AJ, Trivedi MH, Ibrahim HM et al. The 16-Item Quick Inventory of Depressive Symptomatology (QIDS), clinician rating (QIDS-C), and self-report (QIDS-SR): a psychometric evaluation in patients with chronic major depression. Biol Psychiatry 2003; 54(5):573-583.

43. American Psychiatric Association. Diagnostic and Statistical Manual of Mental Disorders. Fourth Edition ed. Washington D.C.: American Psychiatric Association; 1994.
